# Estimating the causal effect of transient anemia status on renal and cardiovascular outcomes in community-dwelling patients in Japan at the beginning of impaired renal function using marginal structural modeling

**DOI:** 10.1007/s10157-021-02137-1

**Published:** 2021-10-01

**Authors:** Satoshi Onozawa, Tomomi Kimura, Yuichiro Ito, Tadao Akizawa

**Affiliations:** 1 Astellas Pharma, Inc, Tokyo, Japan; 2 Astellas Pharma, Inc, 1 Astellas Way, Northbrook, Illinois, 60062 USA; 3 Showa University School of Medicine, Tokyo, Japan

**Keywords:** Anemia, Chronic kidney disease, Marginal structural model

## Abstract

**Background:**

Anemia status may be transient. Causal associations between changes in anemia status over time and adverse outcome development are not well characterized in community-dwelling subjects at the beginning of impaired kidney function.

**Methods:**

This retrospective cohort study used annual health checkup and medical and pharmacy claims data from the JMDC between January 2005 and June 2019. Community-dwelling subjects in Japan with a pre-index estimated glomerular filtration rate (eGFR) ≥ 60 mL/min/1.73 m^2^ followed by a subsequent eGFR < 60 mL/min/1.73 m^2^ (index) were included. The composite renal outcome was ≥ 30% eGFR reduction over 3 years from baseline, serum creatinine doubling, progression to chronic dialysis, kidney transplantation, or eGFR < 15 mL/min/1.73 m^2^. The composite cardiovascular outcome was fatal and non-fatal unstable angina, myocardial infarction, heart failure, or cerebrovascular event. Time-dependent anemia risk was evaluated using Breslow’s estimator and marginal structural Cox models (MSM).

**Results:**

In 32,870 included subjects, 1,396 had anemia at baseline. Adverse outcome incidence was higher in the baseline anemic group, but absolute differences in renal and cardiovascular outcomes between groups were diminished after adjusting for baseline characteristics. In MSM, time-dependent anemia status was associated with higher risk of renal (hazard ratio [95% confidence interval]; 2.6 [1.7–3.8]) and cardiovascular (1.6 [1.2–2.2]) outcomes and mortality (2.8 [1.8–4.3]). Absolute differences in survival probabilities were retained over time but were clinically marginal (1.1–2.7% over 6 years).

**Conclusions:**

Even in subjects at the very early stage of impaired kidney function, early detection and treatment of anemia may help reduce the development of negative sequelae.

**Supplementary Information:**

The online version contains supplementary material available at 10.1007/s10157-021-02137-1.

## Introduction

The prevalence of chronic kidney disease (CKD) in the Japanese adult population is approximately 13%, with most of these individuals in stage 3 [1]. Progression of CKD is associated with increased healthcare costs, decreased patient quality of life, and greater risk for cardiovascular (CV) and all-cause death [2–6].

Anemia of CKD affects approximately 26% of Japanese patients with stage 3 CKD, increasing to greater than half of patients with stage 5 CKD [7]. Unfortunately, because anemia of CKD is frequently asymptomatic, patients often fail to seek medical care, especially during the early stages of CKD [8–13]. Undertreatment of anemia of CKD, particularly in patients with CV diseases and/or DM, is associated with increased rates of blood transfusions, hospitalization, and death [14–16]. Recognition and treatment of anemia of CKD in patients who progress to stage 3 CKD can improve clinical outcomes, including delaying the need for renal replacement therapy [12, 13, 17–19].

Anemia of CKD can be transient in nature, resolving with improvement in CKD management [20]. A post-hoc analysis of the Chronic Renal Insufficiency Cohort (CRIC) study, using marginal structural modeling (MSM) to account for time-dependent confounding, identified an increased risk of incident end-stage kidney disease (ESKD) and death with anemia in mild and moderate CKD in a United States (US) population [21]. However, most prior studies have evaluated the risk of anemia based on the anemia status at baseline [16, 22–25]. The purpose of this study was to determine the causal effect of time-dependent anemia status on renal and CV outcomes and mortality in community-dwelling subjects in Japan at the beginning of impaired renal function. Because anemia status can change over time, and a prior anemia status can affect the potential confounders of the subsequent anemia status (e.g., anemia treatment), the effects of time-varying anemic status was estimated using a counterfactual modeling approach [26]. This model hypothesizes that if a subject were anemic for the entire period of follow-up, the risk of adverse outcomes compared to a subject who was not anemic during the entire period of follow-up would differ.

## Materials and methods

### Study design and data source

This was a retrospective cohort study using annual health checkup data linked to inpatient and outpatient medical claims and pharmacy claims data from the JMDC [27]. The JMDC has over 3 million unique beneficiaries (and their dependents), aged 18–74 years, who were enrolled in one of over 100 Japanese insurance unions. This study obtained approval from the Astellas Medical Affairs Japan Regional Protocol Review Committee (MAJ-PRC) under a unique identifier code or international study number (ISN): 1517-MA-3316.

### Study population and sub-cohorts

Data between January 2005 and June 2019 from subjects with at least two serum creatinine (SCr) measurements were extracted. Estimated glomerular filtration rates (eGFRs) were calculated for each subject using a validated formula for the Japanese population [28]. We identified the first consecutive pair of eGFRs for a subject, within a 2-year timeframe, in which an eGFR ≥ 60 mL/min/1.73 m^2^ was followed by an eGFR < 60 mL/min/1.73 m^2^. Each subject’s first date with an eGFR < 60 mL/min/1.73 m^2^ was defined as the index date. Subjects aged ≥ 18 years as of the index date who had ≥ 1 year of a pre-index lookback period, ≥ 2 years of a follow-up period (unless deceased), and a hemoglobin (Hb) value and dipstick proteinuria examination result at the index date were eligible for inclusion. Subjects were excluded if at least one of these criteria was met: first available eGFR in the database was already < 60 mL/min/1.73 m^2^; all available eGFRs ≥ 60 mL/min/1.73 m^2^; history of chronic dialysis, kidney transplantation, or eGFR < 6 mL/min/1.73 m^2^ at or before the index date; or no eGFR within 38 months after the index date. Subjects were followed up to 30 June 2019 or disenrollment from the JMDC, whichever occurred first.

In addition to the full cohort, two sub-cohorts were evaluated: subjects with a history of CV disease (CV sub-cohort) and subjects who had DM at baseline (DM sub-cohort). A history of CV disease was defined as having previous myocardial infarction, (congestive) heart failure, peripheral vascular disorders, or cerebrovascular disorders, according to International Classification of Diseases, Tenth Revision (ICD10) algorithms for Charlson Comorbidity Index (CCI) [29, 30]. A history of DM was defined according to the CCI ICD-10 algorithms for diagnosis codes of “diabetes, uncomplicated” or “diabetes, complicated,” and/or with antidiabetic treatment/prescription during the 3 months prior to enrollment, or a HbA1c value ≥ 6.5%. The relevant code lists are available in Supplemental Table 1 and Supplemental Table 2.

### Exposure

Anemia was defined by the age-sex specific Hb value according to the “2015 Japanese Society for Dialysis Therapy: Guidelines for Renal Anemia in Chronic Kidney Disease” (Table 1) [31]. In the baseline model, anemia status was determined using the Hb value at index date. In MSM, anemia status was updated using the annual health checkup data.

**Table 1:** Hemoglobin value (g/dL) criteria for anemia definition

	< 60 years old	60–69 years old	≥ 70 years old
Male	< 13.5	< 12.0	< 11.0
Female	< 11.5	< 10.5	< 10.5

### Outcomes

The composite renal outcome was ≥ 30% reduction of eGFR over 3 years from the baseline, SCr doubling, progression to chronic dialysis, receipt of kidney transplantation, or eGFR < 15 mL/min/1.73 m^2^ [5, 32–34]. The composite CV outcome was fatal and non-fatal unstable angina, myocardial infarction (MI), heart failure, or cerebrovascular event (Supplemental Table 1). All-cause death was defined either as a reason for withdrawal from a health insurance plan or recorded as an outcome of the condition.

### Statistical methods

Two models were applied. As a reference model, we assessed the association between baseline anemia status and each outcome of interest (baseline risk model). Subjects were allocated into anemic vs. non-anemic groups, and crude incidence rates for each outcome were calculated. Imbalances between the groups were adjusted using stabilized inverse probability weight (IPW) [35]. IPWs were estimated separately for each CV and DM sub-cohort. Additional details regarding IPW estimation are provided in the Supplemental Methods. The distribution of IPW is provided in Supplemental Fig. 1. In the case of subjects with missing covariates, simple imputation was performed as described in the Supplemental Methods. Unweighted/weighted Kaplan–Meier (KM) estimates were descriptively compared between anemia and non-anemia groups and adjusted hazard ratios (aHRs) were estimated using the Cox proportional hazards model.

Subsequently, we developed an MSM to incorporate the dynamic change in anemia status and factors influencing Hb values during the follow-up. The stabilized IPW was estimated using a pooled logistic model [36]as follows:

1 $$SW_{{ik}} = ~\mathop \Pi \limits_{{k = 0}}^{K} \frac{{P\left( {A_{t} = ~a_{{k,i}} ~|~\overline{A} k - 1 = \bar{a}k - 1,i~} \right)}}{{P\left( {A_{k} = ~a_{{k,i}} ~|~\overline{A} k - 1 = \overline{a} k - 1,i,\overline{C} k = \overline{c} k,i} \right)}}~ ,$$

where i: i^th^ subject.

K: k^th^ day from index date.

$\overline{a} t - 1,i$: Exposure history up to time t-1 of subject i.

$\overline{\mathrm{c} }\text{t,i}$: History of time varying covariates up to time t of subject i.

$\overline{A} - 1$ was defined to be 0. Note in the special case in which k = 0 (at index data), baseline covariates alone were used to estimate IPW.

The time-varying intercept was estimated using a smooth function of the times since index date using natural cubic splines with five knots. To do this, we added three terms as regressors that are specific polynomial functions of time (calculated with the cubic splines SAS Marco RCSPLINE in survrisk.pak, by Frank Harrel, which is publicly available on http://jse.stat.ncsu.edu/70/1s/software/sas). The variables used to estimate the IPW are listed in Supplemental Table 3. We fit the marginal structural Cox model using the IPW estimated with Eq. (1). The robust sandwich variance estimator was used to obtain variance estimates by accounting for the induced correlation among weighted observations [37]. aHRs (anemia vs. no anemia) were estimated using an MSM and survival curves were developed based on the Breslow estimator [38]. Survival probabilities with 95% confidence intervals (CIs) were derived at each year based on the corresponding survival curves. The IPW was estimated separately for the CV and DM sub-cohorts, and aHRs and survival curves were estimated using the same approach.

The slope of eGFR was calculated by fitting a simple linear regression over time. Greedy nearest neighbor one-to-one propensity score matching was performed within a 0.25 caliper. The logit of the propensity score was used in computing differences between pairs of observations. Propensity scores were estimated using logistic regression models. Additional details are provided in the Supplemental Methods.

#### Sensitivity analyses

Two sets of sensitivity analyses were performed for the aHR estimation: (1) treating death as a competing risk for the renal outcomes applying Fine and Gray’s sub-distribution hazard model [39], and (2) replacing weights > 99th percentile with the 99th percentile weight and < 1st percentile with the 1st percentile weight to assess the impact of extreme IPWs. Another sensitivity analysis was performed for estimation of slope of eGFR by restricting the analysis to subjects whose first two post-index eGFR values were < 60 mL/min/1.73 m^2^, as a proxy for stage 3 CKD or worse. Finally, in addition to the MSM using binary anemia status as a time-dependent exposure, another MSM using quintile categories of baseline Hb level was developed by sex to assess the causal effect of categorical Hb levels to the renal outcomes. A multinomial logistic regression model was fitted to estimate weights. The fifth (highest) Hb levels category was set as a reference group.

## Results

### Subject characteristics

Of the 32,870 subjects included in the study, 4,527 and 5,585 comprised the CV and DM sub-cohorts, respectively (Supplemental Fig. 2). Subjects were excluded because they started chronic dialysis before enrollment (*n* = 2), experienced kidney transplantation before enrollment (*n* = 2), had eGFR < 6 ml/min/1.73m^2^ at enrollment (*n* = 5), or had no SCr record within 38 months from the index date (*n* = 900). The earliest index year was 2008. Subject follow-up time is provided in Supplemental Table 4.

The median age was 52 years though < 20% of subjects were aged ≥ 60 years (Table 2). Median eGFRs at pre-index and index were 65 and 58 mL/min/1.73 m^2^, respectively. It was rare for subjects to have underlying conditions associated with anemia development (e.g., malignancy and/or chemotherapy) at baseline. Median Hb at index was 14.8 g/dL, and 4.2% of subjects had anemia at baseline. Additional baseline characteristics are provided in Supplemental Table 5.

**Table 2:** Demographics in total population

		Total (*N* = 32,870)
Age at index (years)	Mean (SD)	51.7 (8.0)
	Median (IQR)	52.0 (46.0–57.0)
	Range (min–max)	21–73
Sex, *n* (%)	Male	23,893 (72.7)
	Female	8,977 (27.3)
eGFR at index (mL/min/1.73m^2^)	Mean (SD)	57.21 (3.00)
	Median (IQR)	58.00 (56.09–59.21)
	Range (min–max)	6.23–59.99
eGFR pre-index (mL/min/1.73m^2^)	Mean (SD)	66.02 (5.25)
	Median (IQR)	64.97 (62.21–68.22)
	Range (min–max)	60.00–139.68
Proteinuria^a^, *n* (%)		2030 (6.2)
Anemia at baseline,^b^ n (%)	Male	937 (3.9)
	Female	459 (5.1)
	Total	1396 (4.2)
Hemoglobin (g/dL)	Mean (SD)	14.7 (1.4)
	Median (IQR)	14.8 (13.8–15.7)
	Range (min–max)	5.7–26.8
Hematocrit (%)	*N*	31,205
	Mean (SD)	44.4 (4.0)
	Median (IQR)	44.5 (41.8–47.0)
	Range (min–max)	17.5–66.7
BMI (kg/m^2^)	*N*	32,865
	Mean (SD)	23.9 (3.7)
	Median (IQR)	23.5 (21.4–25.9)
	Range (min–max)	11.6–55.6
History of CV diseases^c^	*n* (%)	4527 (13.8)
Diabetes^c^	*n* (%)	5585 (17.0)
History of malignancy^d^	*n* (%)	1231 (3.7)
Active malignancy at index	*n* (%)	447 (1.4)
On chemotherapy	*n* (%)	89 (0.3)
History of gastrointestinal hemorrhage^d^	*n* (%)	385 (1.2)
Gastrointestinal hemorrhage at index	*n* (%)	45 (0.1)
Smoking status, *n* (%)	No	24,146 (79.5)
	Yes	6208 (20.5)
Charlson comorbidity index score	Mean (SD)	0.7 (1.15)
	Median (IQR)	0.0 (0.0–1.0)
	Range (min–max)	0–13
Anemia treatment,^e^ *n* (%)	Any	175 (0.5)
	ESA	3 (0.0)
	Oral iron preparation	151 (0.5)
	IV iron preparation	26 (0.1)
	Red blood cell transfusion	15 (0.0)

^a^Dipstick 1 + or above

^b^Defined according to Japanese guidelines by age and sex using Hb value at enrollment

^c^During the entire pre-enrollment period in the database for each subject

^d^During 2 years prior to the enrollment

^e^Defined by prescription during 3 months prior to the enrollment date

*BMI* body mass index, *CV* cardiovascular, *eGFR* estimated glomerular filtration rate, *ESA* erythropoiesis-stimulating agent, *Hb* hemoglobin, *HbA1c* hemoglobin A1c, *IQR* interquartile range, *IV* intravenous, *SD* standard deviation

Compared with the total population, subjects in the CV and DM sub-cohorts were numerically older, more frequently male, more commonly had anemia, and had higher BMIs (Supplemental Table 6). In these sub-cohorts, eGFRs at index were similar but subjects had more proteinuria.

### Baseline characteristics by baseline anemia status

Unweighted and weighted subject characteristics are presented in Table 3. Considerable differences in subjects with anemia at baseline in the unweighted population, such as age, eGFR, proteinuria, HbA1c, smoking status, and CCI score, were balanced after weighting. Anemia was infrequently treated in both groups before and after weighting. Approximately half of the subjects with anemia had an Hb value less than 12 g/dL.

**Table 3:** Weighted and unweighted subject characteristics by status of baseline anemia in total population

*N* (%)		Unweighted			Weighted		
		Anemia at baseline			Anemia at baseline		
		With	Without	SMD ^c^	With	Without	SMD ^c^
Total *N*		1396	31,474		1356	31,482	
Age (years)	Mean (SD)	50.4 (7.1)	51.8 (8.0)	0.1877	51.3 (7.6)	51.7 (8.0)	0.0522
Sex	Male	937 (67.1)	22,956 (72.9)	0.1272	924 (68.2)	22,872 (72.7)	0.0980
	Female	459 (32.9)	8518 (27.1)		431 (31.8)	8610 (27.3)	
eGFR (mL/min/1.73m^2^)	Mean (SD)	55.67 (5.27)	57.28 (2.84)	0.3806	57.19 (3.29)	57.20 (3.14)	0.0008
Proteinuria		161 (11.5)	1869 (5.9)	0.1991	75 (5.5)	1944 (6.2)	0.0287
Hemoglobin (g/dL)	Mean (SD)	11.9 (1.4)	14.9 (1.3)	2.2288	11.9 (1.4)	14.8 (1.3)	2.2387
	< 11 g/dL	360 (25.8)	8 (0.0)	0.8323	365 (26.9)	8 (0.0)	0.8570
Hematocrit (%)	*N*	1314	29,891		1356	31,482	
	Mean (SD)	36.9 (3.7)	44.7 (3.7)	2.1256	37.1 (3.5)	44.7 (3.7)	2.1445
Anemia treatment^a^	Any	54 (3.9)	121 (0.4)	0.2433	8 (0.6)	167 (0.5)	0.0112
	ESA	0	3 (0.0)	0.0138	0	3 (0.0)	0.0147
Oral iron prep		45 (3.2)	106 (0.3)	0.2196	7 (0.5)	145 (0.5)	0.0054
IV iron prep		10 (0.7)	16 (0.1)	0.1078	2 (0.1)	21 (0.1)	0.0232
Red blood cell transfusion		8 (0.6)	7 (0.0)	0.1012	2 (0.1)	10 (0.0)	0.0378
BMI (kg/m^2^)	*n*	1396	31,469		1356	31,482	
	Mean (SD)	22.8 (3.6)	23.9 (3.7)	0.3060	23.4 (3.8)	23.9 (3.7)	0.1317
HbA1c (%)	*n*	1159	25,374		1356	31,482	
	Mean (SD)	5.7 (0.8)	5.6 (0.6)	0.1362	5.6 (0.5)	5.7 (0.6)	0.0035
Smoking status	*n*	1291	29,063		1356	31,482	
	Yes	184 (14.3)	6024 (20.7)	0.1711	268 (19.7)	6404 (20.4)	0.0151
Charlson Comorbidity Index score^b^	Mean (SD)	1.1 (1.79)	0.7 (1.11)	0.3120	0.6 (1.16)	0.7 (1.14)	0.0844

^a^Defined by prescription during 3 months prior to the enrollment date

^b^Comorbidities during the previous year of the enrollment

^c^Absolute standardized mean differences (SMD) were calculated with absolute values > 0.1 considered evidence of meaningful differences

*BMI* body mass index, *eGFR* estimated glomerular filtration rate, *ESA* erythropoiesis-stimulating agent, *HbA1c* hemoglobin A1c, *IV* intravenous, *SD* standard deviation, *SMD* standardized mean difference

**Fig. 1:**
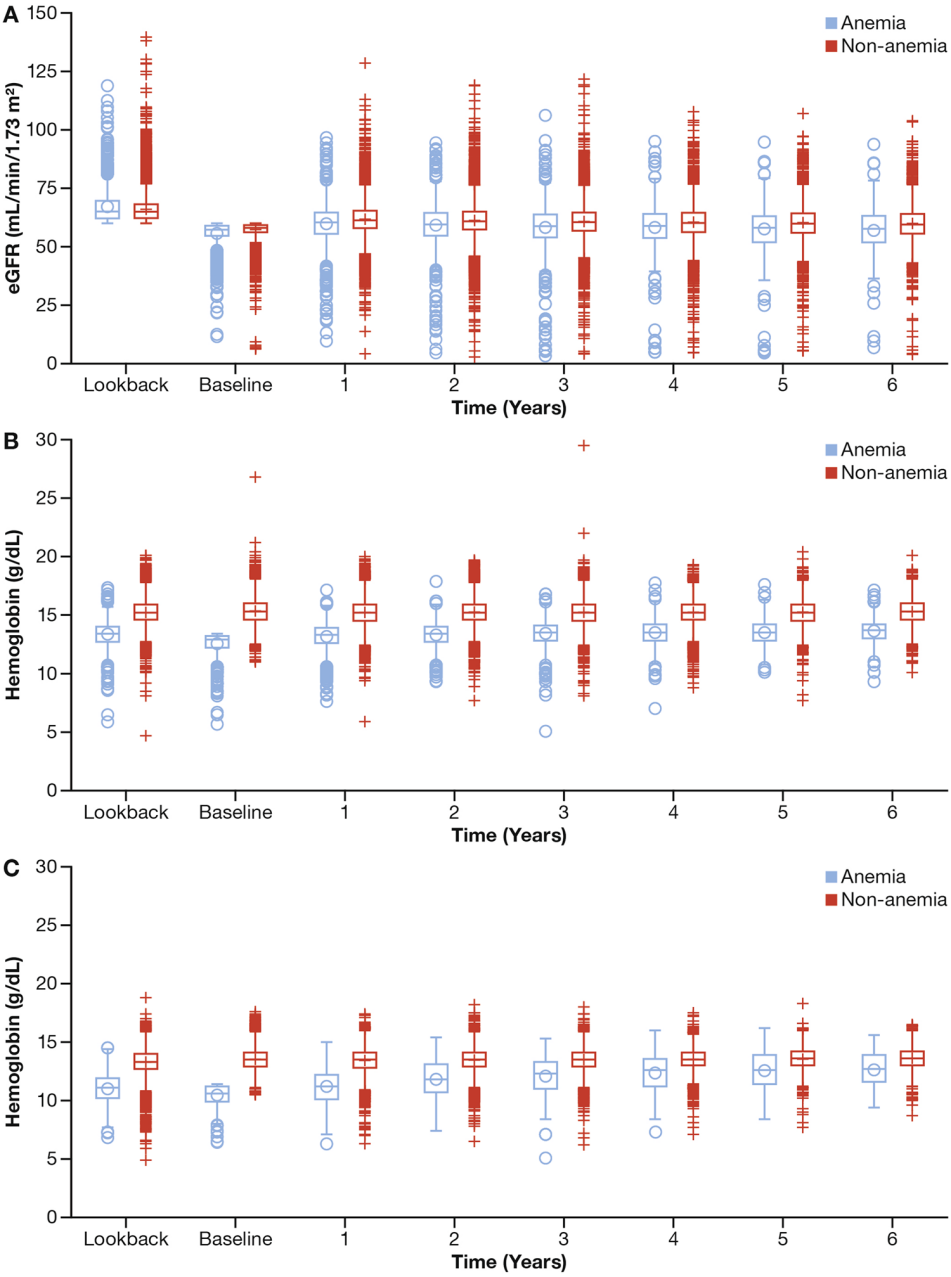
Change in eGFR **a** and hemoglobin values in males **b** and females **c** by anemia status at baseline *eGFR* estimated glomerular filtration rate, *Hb* hemoglobin

The mean (SD) follow-up for the total study cohort was 4.1 (1.85) years. Data are presented through the sixth year because by the third, fourth, and fifth years, the number of subjects with available eGFR and/or Hb values were reduced to one-half, one-third, and one-fifth of their original size, respectively. Mean eGFRs remained at approximately 60 mL/min/1.73 m^2^ throughout the follow-up period regardless of baseline anemia status (Fig. 1A). Hb values in male subjects with anemia at baseline were consistently lower than those in non-anemic subjects (Fig. 1B). While Hb values in female subjects with anemia at baseline gradually increased, the difference between subjects without anemia became smaller over time (Fig. 1C).

### Incidence rate of, and adjusted hazard ratios for, renal and cardiovascular outcomes and mortality

Subjects with anemia at baseline had higher incidence/1000 patient years of mortality and renal and CV outcomes (Table 4). Similar trends were also observed in the CV and DM sub-cohorts (Table 4). The most frequently observed renal outcome was a ≥ 30% decline in eGFR (*n* = 191, 0.58%) (Supplemental Table 7).

**Table 4:** Incidence/1000 patient-years of renal, cardiovascular, and mortality outcomes

Renal outcomes	*N*	Patient-years	Event, *n*	Incidence/1000 patient-years [95% CI]
Total	32,863	135,303.4	210	1.55 [1.35–1.78]
Anemia	1394	5773.2	37	6.41 [4.51–8.83]
Non-anemia	31,469	129,530.2	173	1.34 [1.14–1.55]
CV sub-cohort				
Total	4526	17,870.9	58	3.25 [2.46–4.20]
Anemia	240	936.3	13	13.88 [7.39–23.74]
Non-anemia	4286	16,934.6	45	2.66 [1.94–3.56]
DM sub-cohort				
Total	5583	22,467	120	5.34 [4.43–6.39]
Anemia	310	1166.9	27	23.14 [15.25–33.66]
Non-anemia	5273	21,300	93	4.37 [3.52–5.35]

CV outcomes	*N*	Patient-years	Event, *n*	Incidence/1000 patient-years [95% CI]
Total	32,868	133,497.5	1039	7.78 [7.32–8.27]
Anemia	1396	5692	80	14.05 [11.14–17.49]
Non-anemia	31,472	127,805.5	959	7.50 [7.04–7.99]
CV sub-cohort				
Total	4526	16,996.9	411	24.18 [21.90–26.64]
Anemia	240	866.4	47	54.25 [39.86–72.14]
Non-anemia	4286	16,130.5	364	22.57 [20.31–25.01]
DM sub-cohort				
Total	5585	21,837.9	397	18.18 [16.43–20.06]
Anemia	312	1165.4	37	31.75 [22.35–43.76]
Non-anemia	5273	20,672.5	360	17.41 [15.66–19.31]

Mortality	*N*	Patient-years	Event, *n*	Incidence/1000 patient-years [95% CI]
Total	32,870	135,820.6	250	1.84 [1.62–2.08]
Anemia	1396	5881.8	48	8.16 [6.02–10.82]
Non-anemia	31,474	129,938.7	202	1.55 [1.35–1.78]
CV sub-cohort				
Total	4527	17,991.4	67	3.72 [2.89–4.73]
Anemia	240	964.3	17	17.63 [10.27–28.23]
Non-anemia	4287	17,027.1	50	2.94 [2.18–3.87]
DM sub-cohort				
Total	5585	22,754.9	85	3.74 [2.98–4.62]
Anemia	312	1252.7	20	15.97 [9.75–24.66]
Non-anemia	5273	21,502.2	65	3.02 [2.33–3.85]

*CI* confidence interval, *CV* cardiovascular, *DM* diabetes mellitus

Anemia at baseline, as well as time-dependent anemia status, were independently associated with higher risk of renal, CV, and survival outcomes (Table 5). The MSM analyses showed clearer associations than baseline risk models. In the DM sub-cohort, anemia was not associated with elevated risk for CV outcomes. In the CV sub-cohort, risk of renal outcomes became marginal when competing risk of death was taken into consideration.

**Table 5:** Adjusted hazard ratios for renal and cardiovascular outcomes and mortality

	Total		CV Sub-Cohort		DM Sub-Cohort	
	N*	aHR [95% CI]	N*	aHR [95% CI]	N*	aHR [95% CI]
Renal outcomes						
Baseline risk model						
Primary	32,863	1.943 [1.289–2.928]	4526	2.193 [1.051–4.576]	5583	1.934 [1.189–3.148]
Sensitivity analysis (competing risk with death)^a^		1.744 [1.086–2.802]		2.005 [0.932–4.313]		1.875 [1.096–3.209]
MSM						
Primary	32,863	2.558 [1.709–3.830]	4526	3.182 [1.489–6.803]	5583	1.981 [1.131–3.470]
Sensitivity analysis (competing risk with death)^a^		2.509 [1.676–3.757]		3.145 [1.472–6.719]		1.876 [1.051–3.350]
Sensitivity analysis (weight truncated at 1 and 99 percentile)		3.001 [2.051–4.390]		3.705 [1.779–7.715]		2.486 [1.505–4.106]
CV outcomes						
Baseline risk model	32,868	1.342 [1.052–1.712]	4526	1.476 [1.057–2.063]	5585	1.090 [0.756–1.571]
MSM						
Primary	32,868	1.630 [1.202–2.211]	4526	1.833 [1.082–3.103]	5585	1.469 [0.933–2.311]
Sensitivity analysis (weight truncated at 1 and 99 percentile)		1.709 [1.311–2.228]		1.719 [1.117–2.644]		1.677 [1.095–2.566]
Mortality						
Baseline risk model	32,870	3.440 [2.389–4.955]	4527	3.215 [1.623–6.366]	5585	2.864 [1.533–5.353]
MSM	32,870	2.764 [1.781–4.289]	4527	3.885 [1.592–9.482]	5585	4.155 [2.090–8.261]
Sensitivity analysis (weight truncated at 1 and 99 percentile)		3.646 [2.485–5.347]		4.534 [1.948–10.555]		4.891 [2.484–9.630]

Baseline risk model: Adjusted by baseline age, sex, eGFR, proteinuria, Charlson comorbidity index score, HbA1c, SBP, current smoking, and cardiovascular outcomes (cardiovascular only)

MSM: Adjusted by stabilized inverse probability weight calculated based on the age, sex, and anemia status at time t-1 plus following variables at time t-1 and time t: eGFR, proteinuria, HbA1c, smoking habit, anemia treatment, use of ARB/ACEi, use of other antihypertensive treatment, use of SGLT2i, use of GLP, use of other diabetes treatment, gastrointestinal hemorrhage, chemotherapy, cancer, and Charlson comorbidity index score

*Fine and Gray model was employed. ^a^Death was treated as a competing event

*ACEi* angiotensin-converting enzyme inhibitor, *aHR* adjusted hazard ratio, *ARB* angiotensin II receptor blocker, *CI* confidence interval, *CV* cardiovascular, *DM* diabetes mellitus, *eGFR* estimated glomerular filtration rate, *GLP* glucagon-like peptide-1 receptor agonist, *HbA1c* hemoglobin A1c, *MSM* marginal structural model, *SBP* systolic blood pressure, *SGLT2i* sodium/glucose cotransporter-2 inhibitor

**Fig. 2:**
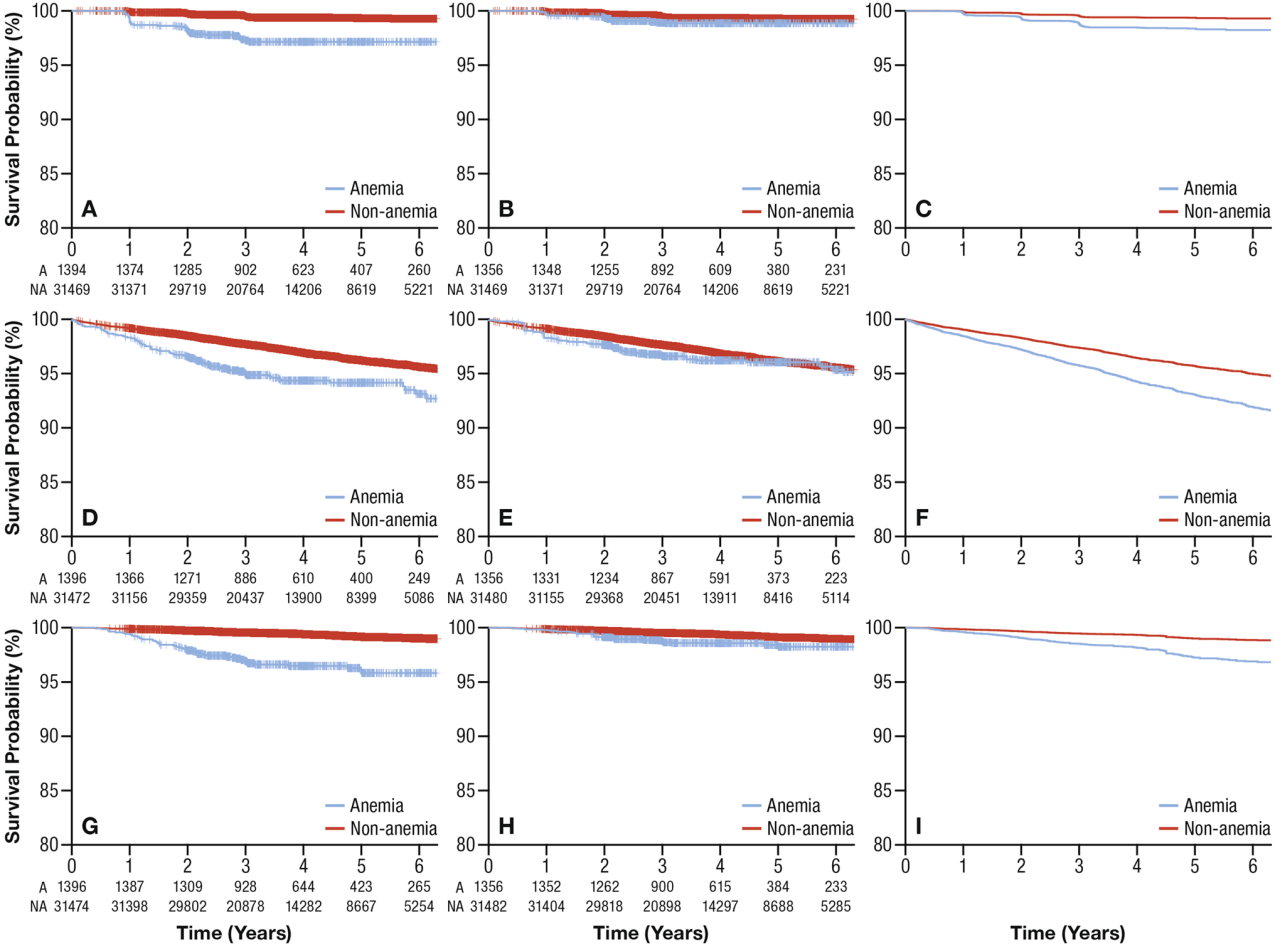
Survival curves for outcomes by anemia at baseline: renal **a** unweighted KM; **b** weighted KM; **c** survival curves from MSM; cardiovascular **d** unweighted KM; **e** weighted KM; **f** survival curves from MSM; mortality **g** unweighted KM; **h** weighted KM; **i** survival curves from MSM Anemic group: blue lines; non-anemic group: red lines. *KM* Kaplan–Meier, *MSM* marginal structural model

Unweighted and weighted KM curves from the baseline risk model and survival curves from the MSM in the entire cohort are shown in Fig. 2**. **Table 6 shows KM estimates and Breslow estimators at Year 1, 3, and 6. Differences between subjects with and without anemia that existed for all three outcomes in the unweighted baseline risk models were minimal and became less apparent after balancing between the groups by weighting. In MSM, differences between subjects with and without anemia were observed for all outcomes, but the absolute differences were also small.

**Table 6:** Survival estimates for renal and cardiovascular outcomes and mortality at 1, 3, and 6 years from the baseline by anemia status

Survival risk (95% CI)	Year 1	Year 3	Year 6
Renal outcomes			
Unweighted Kaplan–Meier			
With baseline anemia	0.9906 (0.9839–0.9945)	0.9726 (0.9620–0.9803)	0.9715 (0.9605–0.9794)
Without baseline anemia	0.9991 (0.9987–0.9994)	0.9950 (0.9941–0.9957)	0.9928 (0.9915–0.9939)
Weighted Kaplan–Meier			
With baseline anemia	0.9967 (0.9864–0.9992)	0.9890 (0.9753–0.9951)	0.9886 (0.9746–0.9949)
Without baseline anemia	0.9991 (0.9987–0.9994)	0.9947 (0.9938–0.9955)	0.9924 (0.9911–0.9935)
Breslow estimator [38]			
With anemia	0.9972 (0.9958–0.9985)	0.9874 (0.9827–0.9921)	0.9823 (0.9756–0.9891)
Without anemia	0.9989 (0.9985–0.9993)	0.9951 (0.9942–0.9959)	0.9930 (0.9918–0.9943)
Cardiovascular outcomes			
Unweighted Kaplan–Meier			
With baseline anemia	0.9835 (0.9753–0.9890)	0.9501 (0.9367–0.9607)	0.9316 (0.9114–0.9473)
Without baseline anemia	0.9922 (0.9911–0.9931)	0.9774 (0.9756–0.9791)	0.9564 (0.9530–0.9595)
Weighted Kaplan–Meier			
With baseline anemia	0.9834 (0.9690–0.9912)	0.9668 (0.9479–0.9789)	0.9539 (0.9261–0.9715)
Without baseline anemia	0.9919 (0.9909–0.9929)	0.9771 (0.9753–0.9787)	0.9559 (0.9525–0.9590)
Breslow estimator [38]			
With anemia	0.9866 (0.9822–0.9910)	0.9631 (0.9521–0.9742)	0.9299 (0.9100–0.9503)
Without anemia	0.9917 (0.9907–0.9928)	0.9772 (0.9755–0.9789)	0.9564 (0.9529–0.9599)
Mortality			
Unweighted Kaplan–Meier			
With baseline anemia	0.9943 (0.9886–0.9971)	0.9694 (0.9584–0.9775)	0.9583 (0.9435–0.9692)
Without baseline anemia	0.9989 (0.9984–0.9992)	0.9955 (0.9946–0.9962)	0.9902 (0.9885–0.9917)
Weighted Kaplan–Meier			
With baseline anemia	0.9979 (0.9876–0.9996)	0.9876 (0.9736–0.9942)	0.9825 (0.9613–0.9921)
Without baseline anemia	0.9988 (0.9983–0.9991)	0.9953 (0.9944–0.9960)	0.9899 (0.9881–0.9914)
Breslow estimator [38]			
With anemia	0.9966 (0.9949–0.9983)	0.9873 (0.9819–0.9926)	0.9732 (0.9623–0.9842)
Without anemia	0.9988 (0.9984–0.9992)	0.9954 (0.9945–0.9962)	0.9902 (0.9881–0.9923)

*CI* confidence interval, *MSM* marginal structural model

For renal outcomes, proteinuria was the most significant risk factor (aHR 7.102, 95% CI 5.216–9.670). Baseline anemia, current smoking status, baseline HbA1c, and CCI score were also positively associated with renal outcomes, and females had lower risk compared with males. For CV outcomes, CV history was the most significant risk factor (aHR 2.503, 95% CI 2.164–2.895). Baseline anemia, proteinuria, CCI score, HbA1c, and current smoking status were also positively correlated with CV outcomes, and females had lower risk compared with males. For mortality, baseline anemia was the most significant risk factor (aHR 3.440, 95% CI 2.389–4.955). Proteinuria, current smoking, and comorbidity score were also positively associated with mortality (Supplemental Fig. 3). When we used quintile categories of baseline Hb level as a time-dependent exposure instead of binary anemia status, a similar causal association was observed for renal outcomes (Supplemental Fig. 4). In males, the lower the Hb, the higher the risk of renal outcomes. In females, when the fifth (highest) Hb level category was set as a reference category, females in all the other categories tended to have a higher risk of renal outcomes (Supplemental Table 8).

The mean slope difference between subjects with or without anemia at baseline was -0.7 mL/min/1.73 m^2^ in the baseline analysis, and -0.9 mL/min/1.73 m^2^ in sensitivity analysis, respectively; slope of eGFR, based on anemia status at baseline, is presented in Supplemental Table 9.

## Discussion

Both anemia at baseline and time-varying anemic status were independent risk factors for renal and CV outcomes and mortality in community-dwelling subjects in Japan at the beginning of impaired renal function. Because a potential time-varying exposure (ie, anemia) status and confounder could have been affected by other time-varying variables (eg, prior treatment) and can subsequently mediate the effect on the outcomes of interest, traditional methods for controlling this confounding may not be adequate [40]. MSM allows the changes of exposure status over time (rather than having fixed status of anemia at the baseline), as well as for the effect from confounding variables that change over time to be accounted for throughout a longitudinal study, better reflecting the actual clinical setting.

In this study, renal outcomes were defined as a composite of ≥ 30% reduction of eGFR over 3 years from the baseline, SCr doubling, progression to chronic dialysis, receipt of kidney transplantation, or eGFR < 15 mL/min/1.73 m^2^. Most 91% of the subjects with renal outcomes experienced eGFR decline. This is a validated surrogate endpoint both in early- and late-stage CKD [5, 32–34], which has been associated with renal replacement therapy use and can be measured in a shorter follow-up period. We confirmed that anemia, defined as a binary variable and along a five-category continuum, was associated with this endpoint in community-dwelling subjects in Japan at the very early stage of renal impairment using real-world data. Additionally, eGFR slope appears to be a reasonable surrogate for clinical endpoints in CKD, which was observed in this study and in a recent meta-analysis [41]. In this study, the mean slope difference between subjects with or without anemia at baseline was  − 0.7 (or  − 0.9 in sensitivity analysis), respectively. A meta-analysis suggested that a 0.75 mL/min/1.73 m^2^/year greater treatment effect on the total eGFR slope was associated with an average 27% lower hazard for the clinical endpoint (95% Bayesian Information Criterion, 20% to 34%). A  − 0.7 (or  − 0.9) mL/min/1.73 m^2^/year eGFR slope can be translated to an average 34% (or 43%) higher hazard for the clinical endpoint. Therefore, the observed difference in the current study may indicate a clinically meaningful acceleration in eGFR decline that is influenced by baseline anemia. Prior studies have observed an association between baseline anemia and CKD progression [21, 42–45]. Saraf et al. also used MSM and observed a strong association between anemia and incident ESKD with longer median follow-up time (7.8 years), corroborating our assertion that anemia causally negatively impacts renal outcomes [21]. However, the difference in survival probability (Breslow estimator) was merely 1.1% at Year 6 (98.2% vs. 99.3%), suggesting the clinical impact of this difference remains uncertain. Proteinuria was the most significant risk factor for renal outcomes, though baseline anemia was also positively associated, which is supported by a community-based cohort study [46]. Because the mechanisms by which anemia affects renal outcomes remain predominately theoretical, these hypothesis-generating findings and interventions to mitigate these negative effects are areas for additional research.

The relative risk of time-varying anemia status on CV outcomes was not as pronounced when compared with mortality or renal outcomes, even in the CV and DM sub-cohorts. This aligns with previous studies showing associations between baseline anemia and increased risk of coronary heart disease, stroke, and all-cause death [4, 47, 48]. The difference in Breslow estimators between anemic and non-anemic groups became larger over time with no difference observed for KM estimates, suggesting that longer periods of being anemic increased the likelihood of CV events.

The higher risk of death observed in subjects with anemia corroborated findings from a previous study in US CKD subjects [45] and a study of almost 63,000 Japanese subjects with varying degrees of renal function [49], but not with a previous study using MSM [21]. This lack of association may have occurred because subjects had higher-stage CKD in the prior study using MSM than in this study, predominately received anemia treatment, and were at greater risk for mortality from other causes that may have resulted in residual and unmeasured confounding.

The current study has potential limitations. Because the censoring and disenrollment from the insurance program may have resulted from renal dysfunction development requiring dialysis initiation and the inability for some subjects to continue to work, the risk of renal outcomes may be underestimated. However, in this cohort, the most frequently observed renal outcome was a ≥ 30% decline in eGFR, and initiation of chronic dialysis was limited, which suggests minimal to no effect from disenrollment because of this scenario. Although missing annual health checkup data may not have occurred completely at random and imputation methods cannot truly compensate for missing values, minimal differences were found when data were compared before and after imputation. Additionally, this study used a validated case definition for myocardial infarction but not for the other CV outcomes. The current study combined diagnosis with specific prescriptions or procedures and diagnosis records in inpatient claims in a thorough and reasonable manner though the validity of individual case definitions is unknown.

## Conclusions

Time-varying anemia status was associated with increased risk of renal and CV outcomes and higher mortality. Because anemia status may be transient and can spontaneously recover at the very early stage of CKD, early detection and treatment of anemia may help delay further decline in renal function and reduce the development of other negative sequelae.

## Supplementary Information

Below is the link to the electronic supplementary material.

Supplementary file1 (DOCX 1310 KB)

## Data Availability

Researchers may request access to anonymized participant level data, trial level data and protocols from Astellas sponsored clinical trials at www.clinicalstudydatarequest.com. For the Astellas criteria on data sharing see: https://clinicalstudydatarequest.com/Study-Sponsors/Study-Sponsors-Astellas.aspx.
