# Supplementary material for: Estimating the causal effect of transient anemia status on renal and cardiovascular outcomes in community-dwelling patients in Japan at the beginning of impaired renal function using marginal structural modeling
Source: Clin Exp Nephrol. 2021 Oct 1;26(2):178–89. doi: 10.1007/s10157-021-02137-1 (PMC8770383; doi:10.1007/s10157-021-02137-1)

**Supplemental Appendix**

**Table of Contents**

Supplemental Methods: pages 2-6

Supplemental Table 1: page 7

Supplemental Table 2: pages 8

Supplemental Table 3: page 9

Supplemental Table 4: page 10

Supplemental Table 5: pages 11

Supplemental Table 6: page 12

Supplemental Table 7: page 13

Supplemental Table 8: page 14

Supplemental Table 9: page 15

Supplemental Figure 1: page 16

Supplemental Figure 2: page 17

Supplemental Figure 3: page 18

Supplemental Figure 4: page 19

**Supplemental Methods**

***Baseline Characteristics***

Diagnosis codes were defined as *International Classification of Diseases 10th Revision* (ICD-10) codes and/or standard disease codes in inpatient or outpatient medical claims. Diagnoses with a suspicion flag were not counted. Charlson Comorbidity Index scores were calculated using the diagnosis codes recorded on the inpatient and outpatient medical claims during the period between the annual health checkups and were updated at each annual health checkup date (almost annually). Data pertaining to pharmaceutical treatment (yes/no) were updated quarterly beginning at the index date. Recent gastrointestinal hemorrhage and active malignancy were used for potential other reason for anemia and updated monthly for any new incidents during the follow-up. Missing values for baseline covariates were imputed with a single imputation and missing in time-varying covariates by last observation carried forward method. Proteinuria was defined as 1+ protein or above from a urine dipstick.

***IPW estimation for balancing between groups with and without baseline anemia***

For an anemic subject *i* ($A_{i}=1$) at baseline with baseline covariates L:

$$W_{i}=\frac{Pr\left[ A_{i}=1 \right]}{Pr\left[ A_{i}=1| L_{i} \right]}$$

For non-anemic subjects, the weights were estimated as:

$$W_{i}=\frac{1-Pr\left[ A_{i}=1 \right]}{1-Pr\left[ A_{i}=1| L_{i} \right]}$$

To calculate the denominators of the equations above, the following variables were included. The continuous variables were age, eGFR, proteinuria, Charlson Comorbidity Index score, HbA1c, systolic blood pressure (SBP), diastolic blood pressure (DBP), and uric acid at baseline. The categorical variables included treatment for anemia, antidiabetic, antihypertensive, antihyperuricemia, gastrointestinal bleeding, cancer, chemotherapy, BMI category (<18.5, 18.5≤, <25, 25≤), current smoking, physical activity, CV history, diabetes history, and enrollment year at baseline. For CV and DM sub-cohorts, IPW was estimated separately for each cohort using the same approach.

***Slope Calculation for Estimated Glomerular Filtration Rate (eGFR)***

To assess the slope of eGFR, eGFR data up to 3 years post- index date were used. Subjects were matched using greedy nearest neighbor one-to-one propensity score matching within a 0.25 calliper. The logit of the propensity score was used in computing differences between pairs of observations. Propensity score was estimated using a logistic regression model. The continuous variables considered included age, eGFR, proteinuria, Charlson Comorbidity Index score, HbA1c, SBP, DBP, and uric acid at baseline. The categorical variables considered were treatment for anemia, antidiabetic, antihypertensive, antihyperuricemia, gastrointestinal bleeding, cancer, treatment for cancer, BMI category (<18.5, 18.5≤, <25, 25≤), current smoking status, physical activity, CV history, diabetes history, and enrollment year at baseline. Slope of eGFR was calculated by fitting a simple linear regression over time. For CV and DM sub-cohorts, slopes of eGFR were estimated using the same approach. A sensitivity analysis was performed for estimation of slope of eGFR by restricting subjects whose first two eGFR measurements were <60 mL/min/1.73 m^2^ (i.e., at baseline and the next measurement) as a proxy for CKD stage G3 or above.

***Missing Values***

Missing values were imputed as follows.

Baseline covariates

Single imputation was performed using the fully conditional specification (FCS) method. The estimation details are as follows.

| Method | Independent variable | Dependent variable |
| --- | --- | --- |
| Regression | Height at baseline | Sex  At baseline: Anemia status, age, treatment for anemia  At look back period: Height, treatment for anemia |
| Regression | SBP at baseline | Sex  At baseline: Anemia status, age, antihypertensive  At look back period: SBP, treatment for hypertension |
| Regression | DBP at baseline | Sex  At baseline: Anemia status, age, antihypertensive  At look back period: DBP, treatment for hypertension |
| Regression | HbA1c at baseline | Sex  At baseline: Anemia status, age, antidiabetic  At look back period: DBP, treatment for diabetes |
| Regression | Urinary acid at baseline | Sex  At baseline: Anemia status, age, antihyperuricemia  At look back period: Urinary acid, treatment for urinary acid |
| Regression | BMI at baseline | Sex  At baseline: Anemia status, age  At look back period: BMI |
| Logistic regression | Proteinuria at baseline | Sex  At baseline: Anemia status, age  At look back period: Proteinuria |
| Logistic regression | Smoking at baseline | Sex  At baseline: Anemia status, age  At look back period: Smoking |

Time-varying covariates

If time-varying covariates were missing, the missing values were imputed by last observation carried forward method.

***Outcomes Definitions and Measurement***

Renal outcomes

Any of the first date of the following was defined as the event.

- ≥30% reduction of eGFR in 3 years from the baseline
- SCr doubling from the baseline
- Initiation of chronic dialysis
  - dialysis procedure at least 3 consecutive months
- The first surgical procedure for kidney transplantation (recipient)
- eGFR value <6 mL/min/1.73 m^2^

Fatal and non-fatal CV outcomes (composite of the following)

Any of the first Treatment Initiation Date after the enrollment date of the following diagnosis in inpatient claims (taken only when it was flagged as “primary diagnosis” or “triggered hospitalization”) was defined as the event. MI diagnosis codes were combined with procedure codes and cerebrovascular events diagnosis were combined with prescription records.

- Unstable angina
- MI
- Heart failure
- Cerebrovascular events

Death

- All-cause death
  - Death was defined either as a reason of withdrawal from health insurance plan or as recorded as an outcome of the condition (diagnosis)

Time-fixed (baseline) covariates

- Sex, history of CV diseases, diabetes or fracture, active malignancy, gastrointestinal hemorrhage, and calendar year of enrollment were used as time-fixed covariates.
- Diagnosis codes were defined as International Classification of Diseases 10^th^ revision (ICD10) codes and/or standard disease code in inpatient (including DPC) or outpatient medical claims. Diagnoses with suspicion flag were not counted.
- Medical history was defined with diagnosis (or treatment) record before or on the enrollment date, unless otherwise specified below. No specific lookback period was set; we looked back as far as possible in the database.
  - CV history was defined as having myocardial infarction, congestive heart failure, peripheral vascular disorders, or cerebrovascular disorders, according to ICD10 algorithms for Charlson Comorbidity Index (CCI).
  - Diabetes was defined as diagnosis codes or antidiabetic treatment or HbA1c values.
    - Diagnosis codes were defined according to the CCI ICD10 algorithm for “diabetes, uncomplicated” and “diabetes, complicated”
    - Antidiabetic prescription record (see Annex)
    - HbA1c level ≥6.5%
  - Fracture history was defined as diagnosis codes listed in Annex 3
  - Gastrointestinal hemorrhage
    - Gastrointestinal hemorrhage history was defined as diagnosis codes during the period between -2 years to -1 month from the enrollment date
    - Gastrointestinal hemorrhage at enrollment was defined as diagnosis during the month of enrollment and if the Treatment Initiation Date was before the enrollment
  - Active malignancy
    - Active malignancy history was defined as diagnosis for cancer and/or chemotherapy in inpatient and outpatient medical or pharmacy claims during the period between -2 years to -1 month from the enrollment date
    - Active malignancy at enrollment was defined as diagnosis or chemotherapy in inpatient and outpatient medical or pharmacy claims during the month of enrollment and if the Treatment Initiation Date was before the enrollment
    - Chemotherapy at enrollment was defined as prescription during the month of enrollment
- The following variables were obtained from annual health checkup data during lookback period (including enrollment date). If multiple measurements were available, use the closest value to the enrollment date (inclusive).
  - Age, eGFR, proteinuria (yes if dipstick was 1+ or above vs no), Hb (g/dL), hematocrit (Ht, %), HbA1c (%), systolic blood pressure (SBP, mmHg), diastolic blood pressure (DBP, mmHg), serum uric acid (UA), body mass index (BMI, kg/m^2^; <18.5 vs. 18.5 < 25 vs. ≥25), current smoking (yes/no), physical activity (yes if ≥1-hour walking or equivalent per day vs. no)
  - eGFR was estimated using the formula for Japanese subjects:

$$eGFR for male=194 \times\mathrm{SCr}^{-1.094} \times\mathrm{age}^{-0.287}$$

$$eGFR for female=194 \times\mathrm{SCr}^{-1.094} \times\mathrm{age}^{-0.287} \times0.739$$

- Charlson Comorbidity Index score was calculated using the diagnosis codes recorded on the inpatient and outpatient medical claims in the 1 year prior to enrollment
- The pharmaceutical treatment (yes/no) was obtained from medical or pharmacy claims during the 3 month-period prior to the enrollment (including enrollment date)
  - Anemia treatment (iron preparation [IV, oral], ESA, red-cell transfusion), antidiabetics (SGLT2i, GLP or others), antihypertensive (ARB/ACEi or others), antihyperuricemia

Time-varying covariates

- The following variables were updated at the time of the annual health checkup during the follow-up period:
  - Age, eGFR, proteinuria, Hb, Ht, HbA1c, SBP, DBP, UA, BMI, current smoking, physical activity
- Charlson Comorbidity Index score was calculated using the diagnosis codes recorded on the inpatient and outpatient medical claims during the period between the annual health checkups, and updated at each annual health checkup date (almost annually)
- The pharmaceutical treatment (yes/no) was updated every quarter from the enrollment date
  - Anemia treatment, antidiabetics, antihypertensive, antihyperuricemia, and chemotherapy

Gastrointestinal hemorrhage and active malignancy were updated monthly for a new incident during the follow-up.

**Supplemental Table 1.** Code list for procedure and diagnosis

| Items | Variable | Code |
| --- | --- | --- |
| Dialysis | Procedure code | J038 (Hemodialysis)  J042 (Peritoneal dialysis) |
| Kidney transplantation (event) | Procedure code | K780 |
| Kidney transplantation (history) | ICD-10 | Z940, T861 |
| Unstable angina | ICD-10 | I20 |
| Myocardial infarction | ICD-10 | I21.x, I22.x |
|  | Procedure code | K546, K547, K548, K549, K550 (Percutaneous Coronary Intervention)  K551, K552 (Coronary Artery Bypass Graft) |
| Heart failure | ICD-10 | I50 |
| Thromboembolism | ICD-10 | G08, I236, I240, I269, I513, I676, I73, I74, I80, I82, K645, M311 |
|  | YJ code (first 7 digits) | 3623033, 8840658, 3623048, 8840636, 8840646, 8840619, 8841669, 8833133, 8837828, 8837827, 8838744, 8838745, 8838742, 8838301, 8838300, 8837622, 8837620, 8836545, 8836543, 8833557, 8833555, 8834813, 8834812, 8838736, 8838705, 8838722, 8839253, 8840693, 8840691, 8847454, 8847494, 8837697, 8837693, 8837695, 8833135, 8835631, 8835629 |
| Cerebrovascular outcomes | ICD-10 | I60, I61, I62, I63, I64 |
| Infection |  | Hospitalizations with anti-infective at the admission date |
| Fracture | ICD-10 | S22: thoracic spine, S32: lumbar spine and pelvis, S42: clavicle and upper arm, S52: forearm, S62: wrist and hand, S72: femur, S82: lower leg, S92: ankle and foot, T10: upper limb, T12: lower limb |
| Gastrointestinal hemorrhage | ICD-10 | K25.0, K25.2, K25.4, K25.6, K26.0, K26.2, K26.4, K26.6, K27.0, K27.2, K27.4, K27.6, K28.0, K28.2, K28.4, K28.6, K29.0, K66.1, K92.2 |
| Active malignancy | ICD-10 | C00-C97 |

*ICD-10* International Classification of Diseases 10^th^ Revision.

Diagnosis with suspicion flag will not be counted. Cardiovascular outcomes were defined as diagnosis codes recorded on inpatient [or Diagnosis Procedure Combination (DPC)] claims and with treatment initiation date after the enrollment date.

**Supplemental Table 2. Code list for prescription**

| Treatment (Anemia) | YJ code | |
| --- | --- | --- |
| Erythropoiesis-stimulating agents | first 4 digits = 3999 and next 3 digits in (412, 427, 413, 425, 432) | |
| Iron preparation | first 4 digits = 3222  Oral: YJ code next 3 digits = 013, 007, 003, 004, 010, 012  IV: YJ code next 3 digits = 400 | |
| Red cell transfusion | first 4 digits = 6342 and next 3 digits in (403, 405, 408, 410, 413, 415), or  Procedure code = K920 | |
| Antihypertensive | first 3 digits = 213, 214  ARB/ACEi and others will be categorized separately as follows:  YJ code first 4 digits = 2149 and  next 3 digits = 110, 111, 112, 113, 114, 115, 116, 117, 118, 119, 120, 121, 122, 039, 040, 041, 042, 044, 046, 048  or  YJ code first 4 digits = 2144 and  next 3 digits = 001, 0A1, 002, 003, 004, 005, 006, 007, 008, 009, 010, 011, 012 | |
| Antidiabetic | first 3 digits = 396 or  first 4 digits = 2492 or  first 4 digits = 2499 and next 3 digits = 410, 411, 415, 416  SGLT2i and GLP-1 receptor agonists will be categorized separately as follows for their reno-protective effect:  ***SGLT2i***  first 4 digits = 3969 and  next 3 digits = 018, 019, 020, 021, 022, 023, 106, 107, 108  ***GLP-1 receptor agonists***  first 4 digits = 2499 and  next 3 digits = 410, 411, 415, 416  or  3969500 | |
| Antihyperuricemia | YJ code first 3 digits = 394 | |
| Chemotherapy | YJ code first 2 digits = 42 | |
| Antibiotics | YJ code first 3 digits = 611, 612, 613, 614, 615, 616, 617, 619, 624  Exception: do not count when only helicobacter pylori eradication medications were prescribed (YJ code first 7 digits = 6199100, 6199101, 6199102, 6199103, 6199104, 6199105, 6131001 + (6149003 OR 6419002) + (2329022 OR 2329023 OR 2329028 OR 2329029) | |
| Antifungal | YJ code first 3 digits = 617 | |
| Antiviral | YJ code first 3 digits = 625 | |
| Stroke medications | Edaravone | YJ code first 7 digits = 1190401 |
|  | Ozagrel sodium (<5d) | YJ code first 7 digits = 3999411 |
|  | Argatroban | YJ code first 7 digits = 2190408 |
|  | Alteplase, Monteplase (<3h) | YJ code first 7 digits = 3959402, 3959407 |
|  | Urokinase (<5d) | YJ code first 7 digits = 3954400 |
|  | Glycerin, mannitol | YJ code first 7 digits = 2190501, 2190400 |

*ACEi* angiotensin-converting enzyme inhibitor, *ARB* angiotensin II receptor blocker, *GLP* glucagon-like peptide-1 receptor agonists, *SGLT2i* sodium/glucose cotransporter-2 inhibitors, *YJ code* Japan specific drug code.

**Supplemental Table 3**. Variables used to estimate inverse probability weight

| At Index Date | |
| --- | --- |
| Numerator of equation (1) | Anemia status at time t_-1_ (defined as 0) |
| Denominator of equation (1) | Sex  At time t_-1_  Anemia status (defined as 0）  At time t  Age, eGFR, proteinuria, HbA1c, smoking, treatment for anemia, antidiabetic, antihypertensive, gastrointestinal hemorrhage, cancer, chemotherapy, Charlson comorbidity index |
| Others | |
| Numerator of equation (1) | Anemia status at time t_-1_ |
| Denominator of equation (1) | Sex  At time t_-1_  Anemia status, eGFR, proteinuria, HbA1c, smoking, treatment for anemia, antidiabetic, antihypertensive, gastrointestinal hemorrhage, cancer, chemotherapy, Charlson comorbidity index  At time t  Age, eGFR, proteinuria, HbA1c, smoking, treatment for anemia, antidiabetic, antihypertensive, gastrointestinal hemorrhage, cancer, chemotherapy, Charlson comorbidity index |

*eGFR* estimated glomerular filtration rate, *HbA1c* hemoglobin A1c.

Supplemental Table 4 Follow-up time

|  | | | Anemia at baseline | | |
| --- | --- | --- | --- | --- | --- |
|  | Total | With | | Without |  |
| n | 32870 | 1396 | | 31474 |  |
| Mean, years (SD) | 4.1 (1.85) | 4.2 (1.99) | | 4.1 (1.84) |  |
| Median, years (Q1-Q3) | 3.8 (2.7-5.1) | 3.9 (2.7-5.2) | | 3.8 (2.7-5.1) |  |
| Range, years (Min-Max) | 0.1-10.6 | 0.4-10.5 | | 0.1-10.6 |  |

*SD* standard deviation.

**Supplemental Table 5**. Additional baseline characteristics in total population

|  |  | Total (N=32,870) |
| --- | --- | --- |
| Index year | 2008-2010 | 1,668 (5.1) |
|  | 2011 | 1,092 (3.3) |
|  | 2012 | 2,397 (7.3) |
|  | 2013 | 3,915 (11.9) |
|  | 2014 | 5,773 (17.6) |
|  | 2015 | 6,482 (19.7) |
|  | 2016 | 7,681 (23.4) |
|  | 2017 | 3,862 (11.7) |
| Data period before enrollment, years | Mean (SD) | 4.57 (3.02) |
|  | Median (Q1-Q3) | 3.81 (2.20-6.14) |
|  | Range (min-max) | 1.00-12.50 |
| Data period before enrollment, N (%) | 1 - <2 years | 6,996 (21.3) |
|  | 2 - <3 years | 5,534 (16.8) |
|  | 3 - <4 years | 4,664 (14.2) |
|  | 4 - <5 years | 4,364 (13.3) |
|  | 5+ years | 11,312 (34.4) |
| Hb level, N (%) | 11+ g/dL | 32502 (98.9) |
|  | <11 g/dL | 368 (1.1) |
| Anemia treatment^a^ | Any | 175 (0.5) |
|  | ESA | 3 (0.0) |
|  | Oral iron preparation | 151 (0.5) |
|  | IV iron preparation | 26 (0.1) |
|  | Red blood cell transfusion | 15 (0.0) |

^a^ Defined by prescription during 3 months prior to the enrollment date.

*ESA* erythropoiesis-stimulating agent, *Hb* hemoglobin, *IV* intravenous, *SD* standard deviation.

**Supplemental Table 6**. Demographics and renal- and anemia-related characteristics in cardiovascular and diabetes mellitus sub-cohorts

|  |  | CV sub-cohort | DM sub-cohort |
| --- | --- | --- | --- |
| Total N |  | 4527 | 5585 |
| Age at enrollment | Mean (SD) | 55.3 (7.7) | 54.9 (7.6) |
|  | Median (Q1-Q3) | 56 (50-60) | 55 (50-60) |
|  | Range (min-max) | 25-73 | 22-73 |
| Age category, N (%) | <40 years | 94 (2.1) | 124 (2.2) |
|  | 40-49 years | 949 (21.0) | 1225 (21.9) |
|  | 50-59 years | 2070 (45.7) | 2549 (45.6) |
|  | 60+ years | 1414 (31.2) | 1687 (30.2) |
| Sex, N (%) | Male | 3393 (75.0) | 4547 (81.4) |
|  | Female | 1134 (25.0) | 1038 (18.6) |
| eGFR at enrollment, | Mean (SD) | 56.86 (3.47) | 56.60 (3.74) |
| ml/min/1.73m^2^ | Median (Q1-Q3) | 57.79 (55.63-59.21) | 57.55 (55.34-59.09) |
|  | Range (min-max) | 9.31-59.99 | 11.38-59.99 |
| eGFR before enrollment, | Mean (SD) | 66.21 (5.63) | 66.43 (5.98) |
| ml/min/1.73m^2^ | Median (Q1-Q3) | 65.09 (62.13-68.30) | 65.22 (62.15-68.57) |
|  | Range (min-max) | 60.00-138.08 | 60.00-138.08 |
| Proteinuria,^a^ N (%) |  | 351 (7.8) | 693 (12.4) |
| Anemia at enrollment,^b^ | Male | 202 (6.0) | 259 (5.7) |
| N (%) | Female | 38 (3.4) | 53 (5.1) |
|  | Total | 240 (5.3) | 312 (5.6) |
| Hb, g/dL | Mean (SD) | 14.6 (1.4) | 14.8 (1.5) |
|  | Median (Q1-Q3) | 14.7 (13.7-15.6) | 14.8 (13.9-15.7) |
|  | Range (min-max) | 5.7-20.1 | 5.7-19.9 |
| Hb level, N (%) | 11+ g/dL | 4482 (99.0) | 5514 (98.7) |
|  | <11 g/dL | 45 (1.0) | 71 (1.3) |
| Hematocrit, % | n | 4317 | 5314 |
|  | Mean (SD) | 44.1 (4.0) | 44.4 (4.0) |
|  | Median (Q1-Q3) | 44.1 (41.6-46.7) | 44.6 (42.0-47.1) |
|  | Range (min-max) | 17.5-66.7 | 17.5-59.0 |
| Anemia treatment ^c^ | Any | 40 (0.9) | 47 (0.8) |
|  | ESA | 1 (0.0) | 2 (0.0) |
|  | Oral iron preparation | 35 (0.8) | 39 (0.7) |
|  | IV iron preparation | 3 (0.1) | 6 (0.1) |
|  | Red blood cell transfusion | 6 (0.1) | 8 (0.1) |

^a^ Dipstick 1+ or above

^b^ Defined according to Japanese guidelines by age and sex using Hb value at enrollment.

^c^ Defined by prescription during 3 months prior to the enrollment date.

*CV* cardiovascular, *DM* diabetes mellitus, *eGFR* estimated glomerular filtration rate, *ESA* erythropoiesis-stimulating agent, *Hb* hemoglobin, *IV* intravenous, *SD* standard deviation.

**Supplemental Table 7.** Components of renal outcomes by anemia status at baseline

|  | | Total (N=32,863)^a^ | Anemia  (n=1,394) | Non-anemia (n=31,469) |
| --- | --- | --- | --- | --- |
| Composite^b^ | | 210 | 37 | 173 |
|  | ≥30% eGFR decline | 191 | 35 | 156 |
|  | eGFR <15 ml/min/1.73m^2^ | 10 | 4 | 6 |
|  | SCr doubling | 35 | 10 | 25 |
|  | Chronic dialysis | 7 | 1 | 6 |
|  | Kidney transplantation | 0 | 0 | 0 |

^a^ Subjects with events (n=7) at enrollment were excluded from the analysis of each endpoint.

^b^ Subject can be counted more than once in the breakdown (but not in the composite) if events occurred on the same date.

*eGFR* estimated glomerular filtration rate, *SCr* serum creatinine.

**Supplemental Table 9.** Hemoglobin level at baseline by sex

|  | | Hemoglobin Category ^a^ | | | | |
| --- | --- | --- | --- | --- | --- | --- |
|  | Overall | 1st | 2nd | 3rd | 4th | 5th |
|  |  |  |  |  |  |  |
| Overall, N | 32870 | 6390 | 6310 | 7098 | 6482 | 6590 |
|  |  |  |  |  |  |  |
| Male, N (%) | 23893 (100.0) | 4668 (19.5) | 4616 (19.3) | 5266 (22.0) | 4539 (19.0) | 4804 (20.1) |
|  |  |  |  |  |  |  |
| Female, N (%) | 8977 (100.0) | 1722 (19.2) | 1694 (18.9) | 1832 (20.4) | 1943 (21.6) | 1786 (19.9) |

^a^ Definition of hemoglobin categories (g/dL): 1st [male: 5.7 to <14.4; female: 6.4 to <12.6], 2nd [male: 14.4 to <15.0; female: 12.6 to <13.2], 3rd [male: 15.0 to <15.6; female: 13.2 to <13.7], 4th [male: 15.6 to <16.2; female: 13.7 to <14.3], 5th [male: 16.2 to 26.8; female: 14.3 to 17.6].

**Supplemental Table 9.** Slope of eGFR over 3 years in propensity score–matched cohort with at least three eGFR measurements

|  | | Anemia at baseline | |
| --- | --- | --- | --- |
|  |  | With | Without |
| Matched population ^a^ | n | 1177 | 1177 |
|  | Mean (SD) | 1.3 (4.20) | 2.0 (3.80) |
|  | Median (Q1-Q3) | 1.1 (-0.7 to 3.2) | 1.6 (-0.2 to 3.5) |
|  | Range | -43.1 | -52.7 |
| Subset of total population ^b^ | n | 539 | 539 |
|  | Mean (SD) | -0.2 (4.00) | 0.7 (3.20) |
|  | Median (Q1-Q3) | -0.1 (-1.9 to 1.9) | 0.7 (-0.9 to 2.5) |
|  | Range | -21.3 to 13.0 | -17.2 to 16.1 |

^a^ One non-anemia subject was matched to one anemia subject using propensity score.

^b^ eGFR at next measurement was <60, in addition to the eGFR at baseline.

*eGFR* estimated glomerular filtration rate, *SD* standard deviation.

**Supplemental Figure 1**

Distribution of inverse probability of treatment weight in the full cohort

Year; time from index date in year.

Weights created between 0 year and 1 year are displayed as year = 1; the same rule was applied to the other time points.

**Supplemental Figure 2**

Study disposition

*CV* cardiovascular, *DM* diabetes mellitus, *eGFR* estimated glomerular filtration rate

Note: Subjects may have been classified into the sub-cohort with cardiovascular disease and the sub-cohort with diabetes as these sub-cohorts were not mutually exclusive.


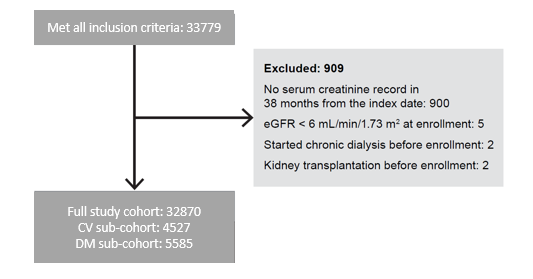


**Supplemental Figure 3**

Risk factors for kidney (a), cardiovascular (b), and mortality outcomes (c)

*CL* control limit, *CV* cardiovascular, *eGFR* estimated glomerular filtration rate, *HbA1c* hemoglobin A1c, *LCL* lower control limit, *SBP* systolic blood pressure, *UCL* upper control limit.


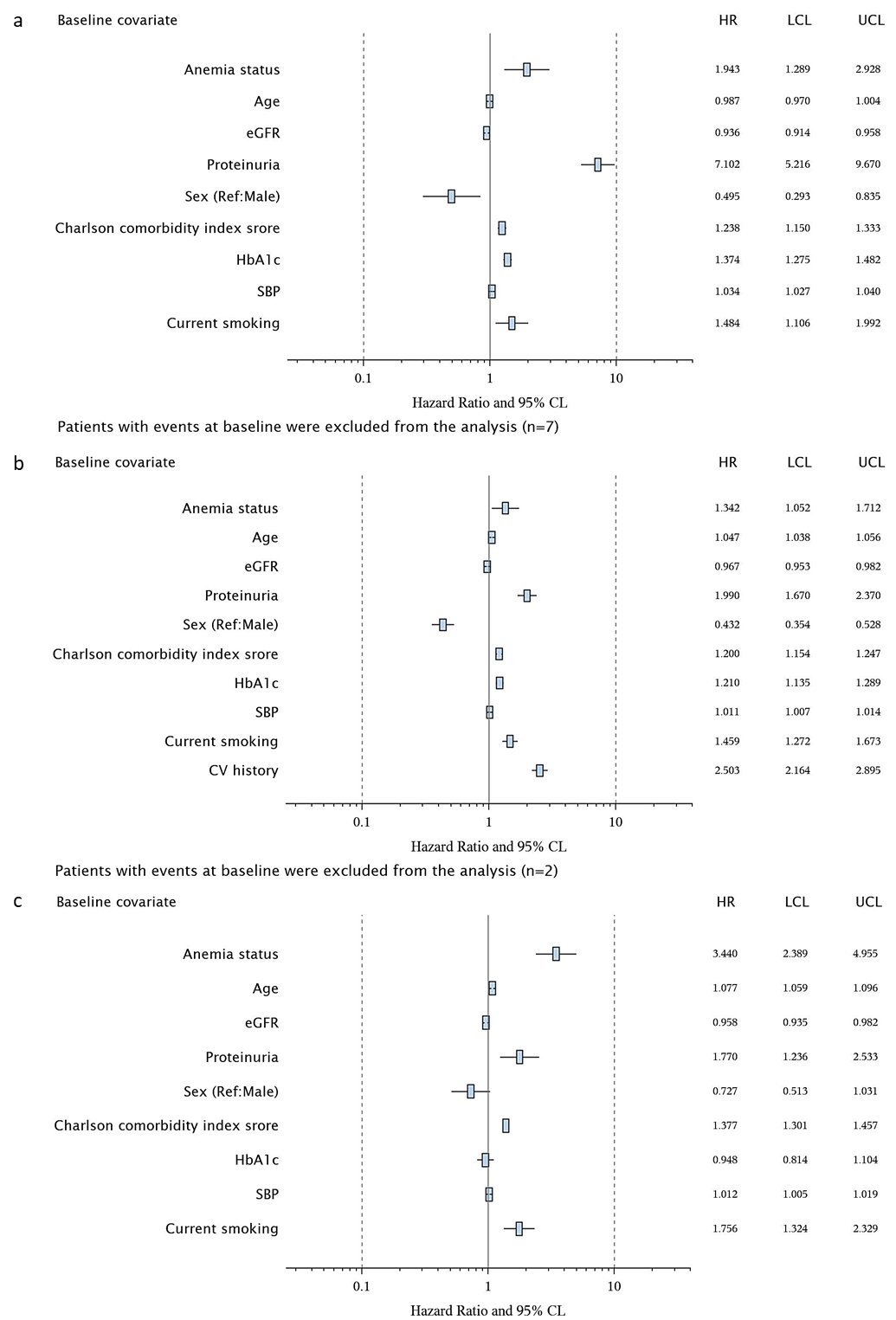


**Supplemental Figure 4**

Hazard ratios from the marginal structural model of the causal effect of time-dependent hemoglobin level on renal outcomes in males (a) and females (b)

*CL* control limit, *Hb* hemoglobin, *LCL* lower control limit, *UCL* upper control limit.

a


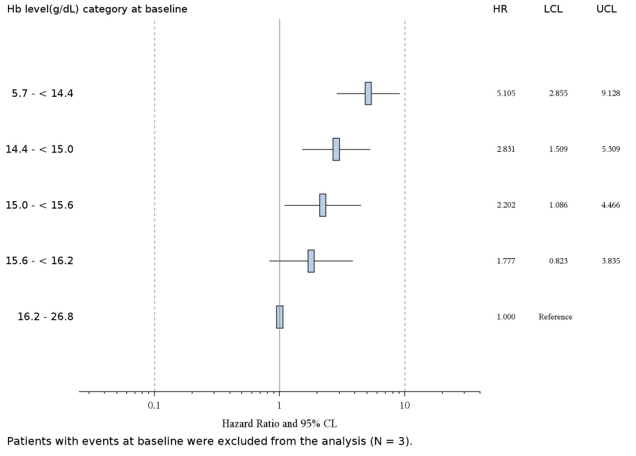


b


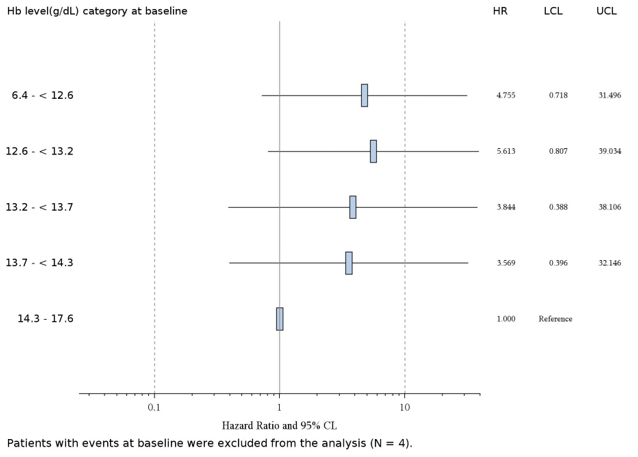

Supplement: Supplementary file 1 — Supplementary file1 (DOCX 1310 KB) [file 10157_2021_2137_MOESM1_ESM.docx]
